# Impaired Gut-Liver-Brain Axis in Patients with Cirrhosis

**DOI:** 10.1038/srep26800

**Published:** 2016-05-26

**Authors:** Vishwadeep Ahluwalia, Naga S Betrapally, Phillip B Hylemon, Melanie B White, Patrick M Gillevet, Ariel B Unser, Andrew Fagan, Kalyani Daita, Douglas M Heuman, Huiping Zhou, Masoumeh Sikaroodi, Jasmohan S Bajaj

**Affiliations:** 1 Gastroenterology, Hepatology and Nutrition, Virginia Commonwealth University and McGuire VA Medical Center, Richmond, Virginia, USA; 2 Microbiome Analysis Center, George Mason University, Manassas, Virginia, USA; 3 Microbiology and Immunology, Virginia Commonwealth University and McGuire VA Medical Center, Richmond, Virginia, USA.

## Abstract

Cirrhosis is associated with brain dysfunction known as hepatic encephalopathy (HE). The mechanisms behind HE are unclear although hyperammonemia and systemic inflammation through gut dysbiosis have been proposed. We aimed to define the individual contribution of specific gut bacterial taxa towards astrocytic and neuronal changes in brain function using multi-modal MRI in patients with cirrhosis. 187 subjects (40 controls, 147 cirrhotic; 87 with HE) underwent systemic inflammatory assessment, cognitive testing, stool microbiota analysis and brain MRI analysis. MR spectroscopy (MRS) changes of increased Glutamate/glutamine, reduced myo-inositol and choline are hyperammonemia-associated astrocytic changes, while diffusion tensor imaging (DTI) demonstrates changes in neuronal integrity and edema. Linkages between cognition, MRI parameters and gut microbiota were compared between groups. We found that HE patients had a significantly worse cognitive performance, systemic inflammation, dysbiosis and hyperammonemia compared to controls and cirrhotics without HE. Specific microbial families (autochthonous taxa negatively and Enterobacteriaceae positively) correlated with MR spectroscopy and hyperammonemia-associated astrocytic changes. On the other hand *Porphyromonadaceae*, were only correlated with neuronal changes on DTI without linkages with ammonia. We conclude that specific gut microbial taxa are related to neuronal and astrocytic consequences of cirrhosis-associated brain dysfunction.

Hepatic encephalopathy (HE) or brain dysfunction due to cirrhosis represents a major healthcare burden in subjects with cirrhosis^1^. Cirrhosis is associated with dysbiosis or an altered gut microbiota that potentiates a systemic pro-inflammatory milieu^2^. This inflammatory environment can potentiate neuro-inflammation, brain edema and ultimately neuronal dysfunction in the setting of hyperammonemia^3^,^4^. However the specific contribution of the gut microbiota towards neuro-inflammation, edema and hyperammonemia in cirrhosis is unclear. Brain imaging in patients with cirrhosis and HE reveals differing impact on neuronal fibers and astrocytes. MR spectroscopy (MRS) findings reveal higher glutamine + glutamate and compensatorily lower myoinositol and choline in astrocytes while diffusion tensor imaging of long white-matter neuronal tracts shows impaired axonal integrity and edema^5^. HE treatments, which mostly act on the gut milieu, have varied impacts on MRS and DTI. While lactulose improves MRS, rifaximin and LOLA do not^6^,^7^,^8^. In contrast rifaximin therapy and lactulose can both favorably impact neuronal integrity as demonstrated by diffusion tensor imaging (DTI)^8^,^9^.

While smaller-scale studies have shown that gut bacteria are associated with cognitive performance as a whole, the linkage between microbial taxa and individual components of the impaired brain response i.e. neuronal and astrocytic impairment is unclear^10^,^11^.

Given the immense complexity of the gut microbiota and their specific modulation with these therapies, the relationship between gut taxa and brain MR consequences becomes relevant in defining focused treatment targets.

The aim of this study was to define the linkage between gut microbial taxa and brain MR consequences of neuronal and astrocytic dysfunction in patients with cirrhosis.

## Results

We recruited 187 subjects for this study; 40 healthy controls and 147 cirrhotics. Eighty-five cirrhotic patients had HE, which was controlled on lactulose and rifaximin. All subjects underwent MRS and 74 subjects underwent DTI. The subjects were age-balanced but controls had a significantly better cognitive performance and lower systemic inflammation than cirrhotic patients (Table 1), especially those with HE.

### Cirrhotic patients and those with HE had worse cognition, inflammation and brain MR findings

On MRS, there were significant differences between controls and cirrhotics on creatine ratios of Glx (WM: 1.7 ± 0.3 vs. 2.5 ± 0.8, p < 0.0001; PGM: 1.9 ± 0.2 vs. 2.3 ± 0.7, p = 0.001; ACC: 2.1 ± 0.3 vs. 2.7 ± 0.7, p = 0.004) and mI (WM: 1.0 ± 0.1 vs. 0.5 ± 0.4, p < 0.0001; PGM: 0.8 ± 0.1 vs. 0.5 ± 0.3, p < 0.0001; ACC: 0.7 ± 0.1 vs. 0.5 ± 0.3, p = 0.001) but not on Choline levels, indicating the impact of hyperammonemia. Cirrhotics with HE were significantly more advanced from the cirrhosis severity and cognitive impairment perspective compared to cirrhotics without HE (Table 1). Using the PHES and ICT cut-offs separately based on our published healthy control data^12^, a significantly higher proportion of prior HE patients had evidence of covert HE by PHES (n = 75, 88% vs. n = 15, 24%,p < 0.001) and by ICT (n = 68, 80% vs n = 20, 32%).

Cirrhotics with HE subjects had higher Glx and lower mI in all three volumes of interest and reduced Cho in all volumes apart from gray matter. In the 74 subjects who underwent DTI, 42 patients had prior HE and had similar overall demographic and cirrhosis severity characteristics compared to the entire HE group. HE patients had a higher CS and lower FA compared to no-HE patients (Table 2). No changes in MD between groups were identified.

### Cirrhotics, especially HE patients, demonstrated dysbiosis and altered microbial functionality

**Figure 1:**
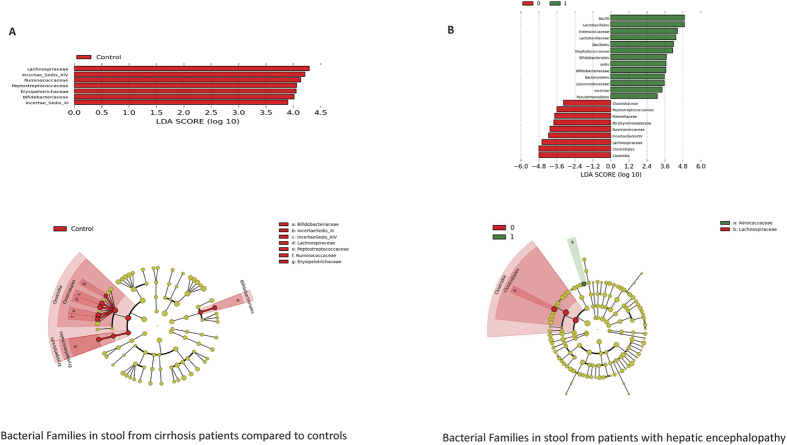
Microbiota analysis of stool 1(**A**). LEfSe predictions for bacterial families found in stool for healthy controls compared to cirrhosis. The histogram shows bacterial families in red bars that were significantly higher in control stool microbiota. LDA (linear discriminant analysis) score on the x-axis represents log changes in relative bacterial family representation in healthy controls compared to cirrhotic patients. The cladogram shows the phylogenetic relationship between the bacterial families that were higher in controls compared to cirrhotic patients that are represented in the red in the histogram. 1(**B**) LEfSe predictions for bacterial families found in stool for patients with hepatic encephalopathy. The histogram shows bacterial families in green bars that were significantly higher in cirrhotics with HE while those in red show bacterial families that were significantly higher in cirrhotics without HE. LDA (linear discriminant analysis) score on the x-axis represents log changes in relative bacterial family representation between the two groups. The cladogram shows the phylogenetic relationship between the bacterial families that were different in cirrhotics with HE compared to cirrhotics without HE representing the same colors for the groups as in the histogram.

As expected there were significant differences in the microbiota composition between cirrhotics with and without HE as well as between controls and cirrhotic subjects on UNIFRAC (all p values < = 1.0e − 02). Specifically controls had a significantly higher proportion of autochthonous bacterial families than cirrhotics (Fig. 1A). Similarly cirrhotic patients with HE had a higher relative abundance of these autochthonous families and a higher abundance pattern of *Staphylococcaceae, Enterococcaceae, Porphyromonadaceae* and *Lactobacillaceae* compared to controls and cirrhotics without HE(Table 3/Fig. 1B). On PiCRUST, significant differences in predicted stool bacterial functionality was found related to functions of endotoxin and endotoxin-protein synthesis and a shift towards an ammoniagenic amino acid profile (degradation of branched-chain and metabolism of aromatic amino acids) in controls compared to cirrhotics and in HE patients compared to no-HE cirrhotic patients (Supplementary Fig. 1A,B).

### Brain MRS and DTI are correlated with different gut microbial families

**Figure 2:**
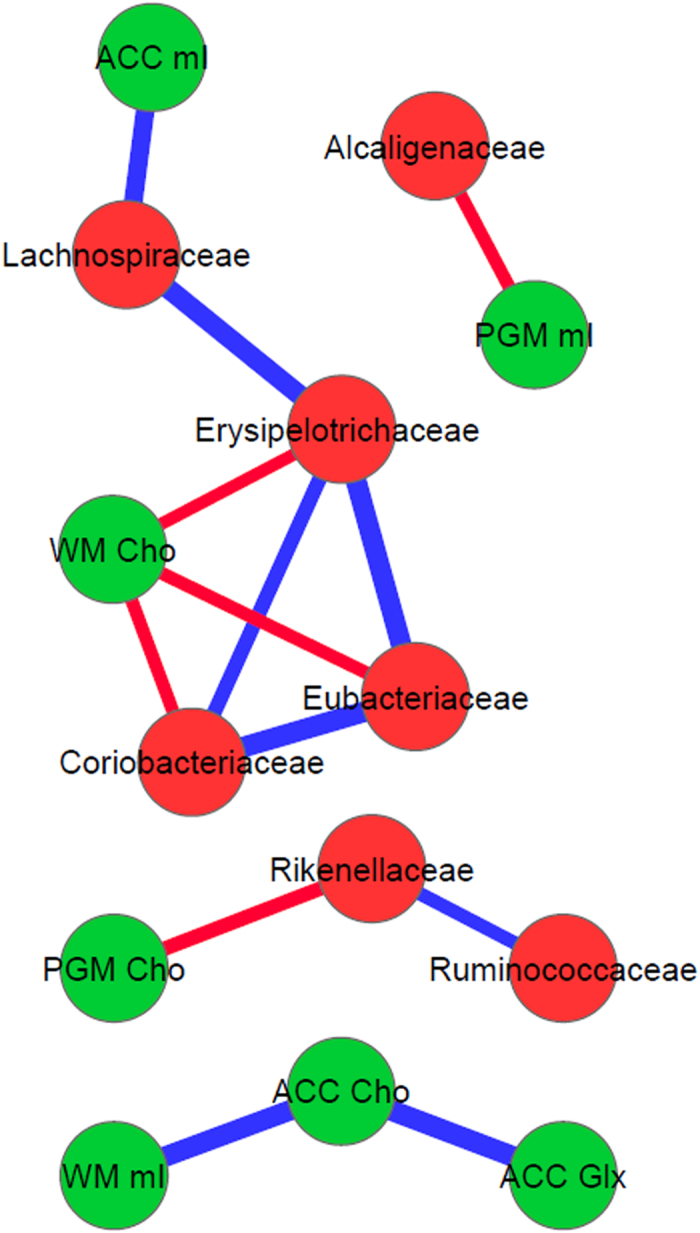
Correlation networks of microbiota, cognitive tests and brain MRS values in controls. Red nodes: stool bacterial families, light green: cognitive test results, dark green: brain MRS values, blue nodes: serum results. Red connections between nodes indicate negative correlation while blue connections are positive linkages. WM: right parietal white matter, PGM: posterior gray matter, ACC: anterior cingulate cortex, Cho: creatine ratio of choline, mI: creatine ratio of myoinositol, Glx: creatine ratio of glutamate + glutamine, BDT: block design test, DST: digit symbol test, NCT-A: number connection test-A, NCT-B: number connection test-B, Dotting: serial dotting test, LTT: line tracing test, targets and lures: outcomes of inhibitory control test. A high score on targets, digit symbol and block design indicates good cognitive performance while a high score on the rest of the cognitive tests suggests the opposite. Control network: There are few connections but the autochthonous family, *Lachnospiraceae* is positively linked with ACC mI and *Alcaligenaceae*, member of Proteobacteria is linked negatively to mI in PGM.

**Figure 3:**
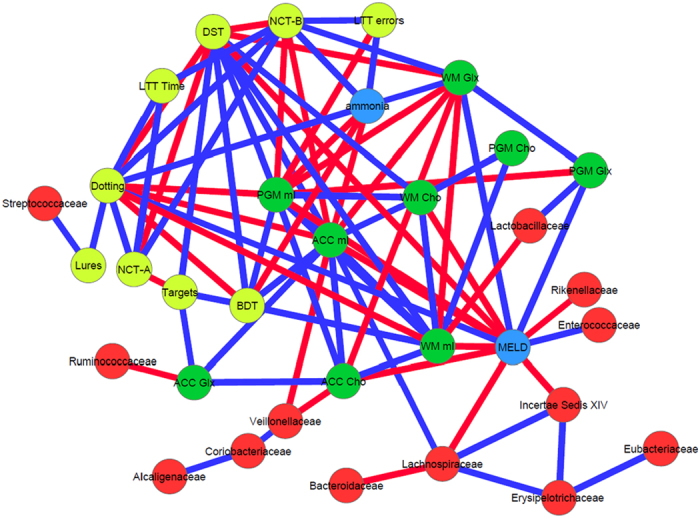
Correlation networks of microbiota, cognitive tests and brain MRS values in all cirrhotic patients. Red nodes: stool bacterial families, light green: cognitive test results, dark green: brain MRS values, blue nodes: serum results. Red connections between nodes indicate negative correlation while blue connections are positive linkages. WM: right parietal white matter, PGM: posterior gray matter, ACC: anterior cingulate cortex, Cho: creatine ratio of choline, mI: creatine ratio of myoinositol, Glx: creatine ratio of glutamate + glutamine, BDT: block design test, DST: digit symbol test, NCT-A: number connection test-A, NCT-B: number connection test-B, Dotting: serial dotting test, LTT: line tracing test, targets and lures: outcomes of inhibitory control test. A high score on targets, digit symbol and block design indicates good cognitive performance while a high score on the rest of the cognitive tests suggests the opposite. Cirrhosis network: In all cirrhosis subjects, there were expected positive linkages between Glx, MELD and ammonia and negative between these and mI and Cho. These were related to poor cognitive performance. Autochthonous taxa (*Lachnospiraceae, Ruminococcaeae* and Incertae sedis XIV) were negatively correlated with MELD score and Glx while potentially pathogenic *Enterococcaceae* were positively related to MELD.

**Figure 4:**
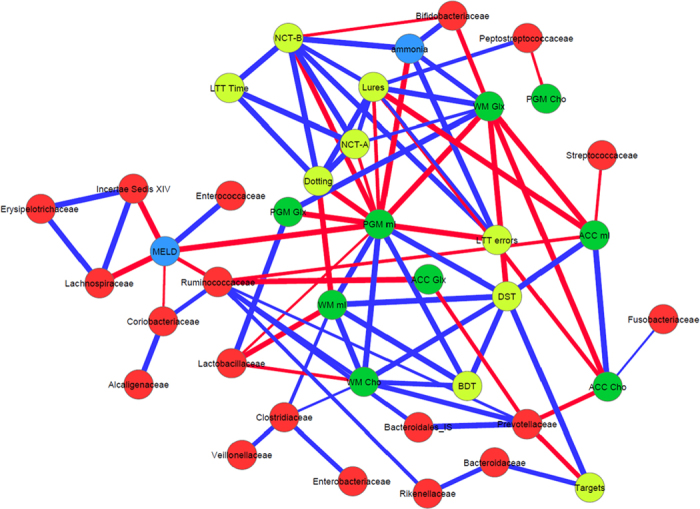
Correlation networks of microbiota, cognitive tests and brain MRS values in cirrhotic patients with HE. Red nodes: stool bacterial families, light green: cognitive test results, dark green: brain MRS values, blue nodes: serum results. Red connections between nodes indicate negative correlation while blue connections are positive linkages. WM: right parietal white matter, PGM: posterior gray matter, ACC: anterior cingulate cortex, Cho: creatine ratio of choline, mI: creatine ratio of myoinositol, Glx: creatine ratio of glutamate + glutamine, BDT: block design test, DST: digit symbol test, NCT-A: number connection test-A, NCT-B: number connection test-B, Dotting: serial dotting test, LTT: line tracing test, targets and lures: outcomes of inhibitory control test. A high score on targets, digit symbol and block design indicates good cognitive performance while a high score on the rest of the cognitive tests suggests the opposite. In HE patients, there was evidence of a robust correlation network in which autochthonous bacterial families, *Ruminococcaeae* and Incertae sedis XIV were negatively correlated with MELD score and to MRS findings of increased Glx. Ammonia was again negatively related to good cognition and positively with Glx. Families such as *Streptococcacae, Lactobacillaceae* and *Peptostreptococcaceae* were related to neuro-inflammation and poor cognition.

**Figure 5:**
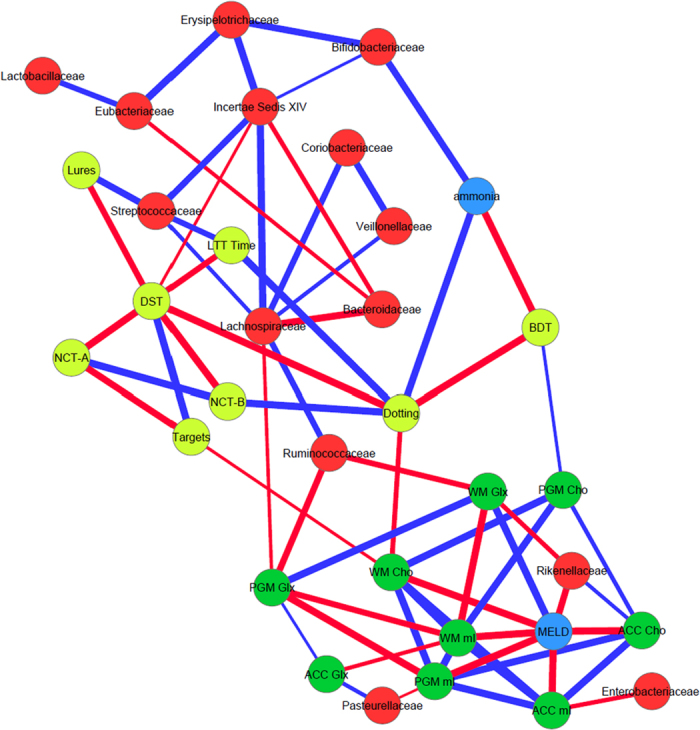
Correlation networks of microbiota, cognitive tests and brain MRS values in cirrhotic patients without HE. Red nodes: stool bacterial families, light green: cognitive test results, dark green: brain MRS values, blue nodes: serum results. Red connections between nodes indicate negative correlation while blue connections are positive linkages. WM: right parietal white matter, PGM: posterior gray matter, ACC: anterior cingulate cortex, Cho: creatine ratio of choline, mI: creatine ratio of myoinositol, Glx: creatine ratio of glutamate + glutamine, BDT: block design test, DST: digit symbol test, NCT-A: number connection test-A, NCT-B: number connection test-B, Dotting: serial dotting test, LTT: line tracing test, targets and lures: outcomes of inhibitory control test. A high score on targets, digit symbol and block design indicates good cognitive performance while a high score on the rest of the cognitive tests suggests the opposite. No-HE patients showed similar correlations between autochthonous taxa and good cognition and lesser neuro-inflammation while members of Proteobacteria such as *Enterobacteriaceae* and *Pastuerellaceae* were linked with worse neuro-inflammation as were *Streptococcaceae*.

On MRS, there were consistent negative linkages between autochthonous families and positive ones between potentially pathogenic ones to brain consequences of hyperammonemia i.e. increased Glx and lower mI and Cho, in controls and cirrhotic patients (Figs 2, 3, 4, 5). These brain changes were also linked with MELD score and serum ammonia levels. These interactions were significantly more complex in patients with HE in whom the non-autochthonous families were positively linked with poor cognition, brain MRS findings and ammonia.

On DTI, a different picture emerged with respect to the families. The family *Porphyromonadaceae* was linked with all aspects of DTI. On FA, *Porphyromonadaceae* relative abundance was correlated negatively with corpus callosum splenium (r = −0.5, p = 0.01), right inferior longitudinal fasciculus (r = −0.4, p = 0.05), posterior internal capsule (left r = −0.5, p = 0.01, right r = −0.5, p = 0.02), posterior white matter (left r = −0.5, p = 0.01, right p = 0.6, p=0.004). In contrast, *Prevotellaceae* relative abundance was positively related with right posterior white matter (r = 0.5, p = 0.04). On spherical isotropy, we found positive correlations between *Porphyromonadaceae* and corpus callosum splenium (r = 0.5, p = 0.009), right inferior longitudinal fasciculus (r = 0.4, p = 0.04) and posterior white matter (left r = 0.45, p = 0.03, right r = 0.6, p = 0.005). There was a significant positive correlation on mean diffusivity between *Porphyromonadaceae* on corpus callosum genu (r = 0.44, p = 0.03), corpus callosum splenium (r = 0.58, p=0.003), left and right posterior white matter (r = 0.7, p < 0.001 for both), left and right frontal white matter (r = 0.4,p = 0.05 for both)and the right uncinate fasciculus (r = 0.4, p = 0.05). Similarly significant positive correlations were found between *Veillonellaceae* and mean diffusivity in the bilateral anterior internal capsule (r = 0.4, p = 0.03), corpus callosum splenium (r = 0.4, p = 0.04), right cingulum (r = 0.5, p = 0.03), external capsule (left r = 0.5, p = 0.03, right r = 0.4, p = 0.05), posterior internal capsule (left r = 0.5, p = 0.02, right r = 0.4, p = 0.05)and the right uncinate fasciculus (r = 0.5, p = 0.004).

## Discussion

The current study is the largest experience of the altered gut-liver-brain axis in humans with cirrhosis. We found that specific gut microbial changes are linked with systemic inflammation, ammonia and ultimately with neuronal and astrocytic dysfunction in cirrhotic patients, especially those with HE. Although there is mounting evidence from human and animal studies that gut microbiota modification can impact brain function in cirrhosis, specific linkages between brain MRI and individual bacterial taxa have not been fully elucidated.

While therapies for HE are overwhelmingly gut-based, there is often an additive role for synergistic, systemic ammonia-scavenging treatments in humans^1^. There is patho-physiologic evidence supporting both hyperammonemia and inflammation in the causation of HE, with differing effects on neurons and astrocytes^13^,^14^. It has been hypothesized that in humans the dysbiotic gut microbiota is the major source of both ammonia and the systemic pro-inflammatory milieu^15^. Specifically with the progression of cirrhosis, the relative reduction in autochthonous commensals and the increase in microbiota such as those belonging to *Enterobacteriaceae* and *Streptococcaceae* that can produce endotoxin and ammonia through their urease activity respectively^11^. We found indeed that in the human context, the brain MRS, which is largely related to hyperammonemia-associated astrocytic changes, was related to one set of microbiota while brain DTI, which is related to neuronal integrity and edema, was related to another group of bacteria^8^. However in patients with HE there is concurrent hyperammonemia and systemic inflammation, therefore differentiating their individual impact on brain MRI results is not possible. The microbial families that were negatively linked with brain glial MRS manifestations of ammonia (high Glx and low mI and Cho) were autochthonous families (*Lachospiraceae, Ruminococcaeae* and Clostridiales XIV). In contrast families such as *Streptococcacae, Enterobacteriaceae, Lactobacillaceae* and *Peptostreptococcaceae* were related positively with ammonia, MELD score and brain MRS manifestations. These autochthonous taxa are predominant in healthy control studies and mediate several important benefits, such as production of short-chain fatty acids and 7-α de-hydroxylation of bile acids in hosts^16^,^17^. With the progression of cirrhosis in humans, their reduction parallels an increase in potentially harmful taxa such as *Streptococcacae* and *Enterobacteriaceae*^2^.

Interestingly, taxa that were associated with DTI were distinct i.e. *Porphyromonadaceae* relative abundance, from that associated with MRS. Relative abundance of *Porphyromonadaceae* mirrors a situation of increasing diffuse white matter interstitial edema that is often seen in HE, due to negative associations with FA and positive ones with spherical isotropy and mean diffusivity^6^. *Porphyromonadaceae* have been implicated in cognitive dysfunction and progression of fatty liver disease in prior human and animal studies^10^,^18^. They are predominantly oral in origin but have been implicated in systemic and hepatic inflammation in animal studies that extend beyond the local oral milieu^18^,^19^. Our findings now associate this family with neuronal dysfunction and brain edema. Of note *Porphyromonadaceae* abundance was not related to ammonia in this dataset. We have reported earlier in a smaller sample size that cognitive dysfunction in cirrhosis is related with decrease in autochthonous families and increase in *Porphyromonadaceae*^10^. However our aggregate results indicate that the individual impact of microbial families may be mediated through impact on different cell types i.e. neurons and astrocytes, in the ultimate pathogenesis of HE-related cognitive dysfunction.

Despite prior smaller studies linking cognition with stool microbiota, this is the largest experience linking gut microbiota with brain MRI results that gives us an understanding of the impaired gut-liver-brain axis in cirrhosis. This is an association study that helps in hypothesis generation, specifically related to the differences in linkages between bacteria related and unrelated to ammonia in the pathogenesis of cognitive dysfunction. Given that this was a clinical sample, all of our patients were on treatment with lactulose or rifaximin. However, it would have been unethical to withdraw this therapy and in a cross-sectional manner, use of these medications did not appreciably reduce systemic inflammation or endotoxemia. We also used the clinically reproducible definition of prior HE instead of covert HE definitions that are variable based on gold standards^1^,^12^. Also as expected, most prior HE patients indeed had evidence of covert HE. It is also interesting that prior HE treatment studies are varied in their impact on DTI and MRS based on the agent employed, however concurrent microbiota analysis was not performed in most studies^7^,^8^,^9^. Therefore linking changes in microbiota with changes in brain MRI over time is needed in further studies.

Interestingly, *Lactobacillaceae*, which often have probiotic species, had an increased relative abundance in the stool of HE patients. It was assumed in past human studies that the increased *Lactobacillaceae* abundance was due to the use of lactulose for HE therapy^20^. However, our prior and current human results and prior animal CCL4 studies demonstrate that *Lactobacillaceae* increase may be a part of an expansion of selected urease-producing Firmicutes in humans and mouse cirrhosis models^2^,^21^,^22^. An increased cerebral lactate, which has now been hypothesized to be synergistic with glutamine for HE development in some animal models, could also be precipitated by *Lactobacillaceae* spp^23^.

We conclude that specific bacterial taxa are associated with astrocytic changes and neuronal changes on brain MRI in humans with cirrhosis and HE. Specific alterations, which provide potential novel therapeutic targets to restore intestinal and neuronal homeostasis, in the gut microbial milieu could impact different aspects of brain function in cirrhotic individuals.

## Materials and Methods

Outpatients with cirrhosis and age-matched healthy controls were recruited prospectively from Liver Clinics and the community at Virginia Commonwealth University and McGuire VA Medical Centers. Cirrhosis was diagnosed using biopsy, history of frank decompensation (ascites, HE, variceal bleeding, jaundice) or varices in a patient with chronic liver disease or those with radiological features of cirrhosis^24^. We excluded subjects who were unable to provide informed consent, those with a mini-mental status exam result <25, those who had an uncertain diagnosis of cirrhosis, those with recent (<6 months history) of alcohol or illicit drug misuse and those on absorbable antibiotics within the last 6 weeks. We only included those with prior type C HE who were controlled on lactulose or rifaximin for at least 3 months with good adherence per clinic notes and interview before enrollment^1^. Healthy controls were subjects without chronic diseases who were not receiving any regular medications.

All subjects gave written informed consent and underwent a cognitive assessment consisting of a standard paper-pencil battery of psychometric hepatic encephalopathy score (PHES, number connection tests A and B, digit symbol, serial dotting and line tracing tests) and the inhibitory control test (lures and targets are outcomes)^25^. Cirrhotic patients were also divided into covert HE or not using 2 gold standard tests (PHES and ICT) based on our published control performance^12^. Blood was drawn for analysis of venous ammonia, endotoxin, inflammatory cytokines (IL-1β, TNF-α, and IL-6) and the MELD score (validated logarithmic index of serum bilirubin, creatinine and INR)^26^. Endotoxin and cytokine analysis was performed using validated methods at Assaygate (Ijamsville, MD)^27^. Subjects were asked to provide a fresh stool sample for analysis of microbiota relative abundance in a container with RNAlater as described in prior studies^28^. This protocol was approved by the Institutional Review Boards at VCU Medical Center and Richmond VA Medical Center and all research activities were carried out in accordance with approved guidelines.

### Sample analysis

#### Microbiota

Stool was collected and DNA extracted using published techniques^27^. We first used Length Heterogeneity PCR (LH-PCR) fingerprinting of the 16S rRNA to rapidly survey our samples and standardize the community amplification. We then interrogated the microbial taxa associated using Multitag Pyrosequencing (MTPS)^29^. This technique allows the rapid sequencing of multiple samples at one time. Microbiome Community Fingerprinting: About 10 ng of extracted DNA was amplified by PCR using a fluorescently labeled forward primer 27F (5′-(6FAM) AGAGTTTGATCCTGGCTCA G-3′) and unlabeled reverse primer 355R′ (5′-GCTGCCTCCCGTAGGAGT-3′). Both primers are universal primers for bacteria. The LH-PCR products were diluted according to their intensity on agarose gel electrophoresis and mixed with ILS-600 size standards (Promega) and HiDi Formamide (Applied Biosystems, Foster City, CA). The diluted samples were then separated on a ABI 3130xl fluorescent capillary sequencer (Applied Biosystems, Foster City, CA) and processed using the Genemapper™ software package (Applied Biosystems, Foster City, CA). Normalized peak areas were calculated using a custom PERL script and operational taxonomic units (OTUs) constituting less than 1% of the total community from each sample were eliminated from the analysis to remove the variable low abundance components within the communities.

#### MTPS

Specifically, we have generated a set of 96 emulsion PCR fusion primers that contain the 454 emulsion PCR linkers on the 27F and 355R primers and a different 8 base “barcode” between the A adapter and 27F primer. Thus, each fecal sample was amplified with unique bar-coded forward 16S rRNA primers and then up to 96 samples were pooled and subjected to emulsion PCR and pyrosequenced using a GS-FLX pyrosequencer (Roche). Data from each pooled sample were “deconvoluted” by sorting the sequences into bins based on the barcodes using custom PERL scripts. Thus, we were able to normalize each sample by the total number of reads from each barcode. We have noted that ligating tagged primers to PCR amplicons distorts the abundances of the communities and thus it is critical to incorporate the tags during the original amplification step^29^.

#### Microbiome Community Analysis

We identified the taxa present in each sample using the Bayesian analysis tool in Version 10 of the Ribosomal Database Project (RDP10). The abundances of the bacterial identifications were then normalized using a custom PERL script and genera present at >1% of the community were tabulated. We chose this cutoff because of our *a priori* assumption that genera present in <1% of the community vary between individuals and have minimal contribution to the functionality of that community and 2,000 reads per sample will only reliably identify community components that are greater than 1% in abundance.

#### Analysis of microbiota

QIIME analysis, LEFSe and Kruskal-Wallis tests were used to evaluate changes in overall microbial abundance^30^. We also performed Metastats to evaluate changes in relative abundance between groups with correction for the false discovery rate (FDR)^31^.Predicted bacterial functions were then assessed using PiCRUST (phylogenetic investigation of communities by reconstruction of unobserved states)^32^.

### Brain MRI evaluation

A subset then underwent multi-modal brain MRI assessment on the day of the cognitive testing and sample collection. All images were acquired on a 3T GE Signa scanner using a quadrature birdcage RF head coil. We tested two strategies to study the impact of cirrhosis in astrocytes and neurons^8^,^33^. For the impact of hyperammonemia-associated astrocytic changes, MR spectroscopy was performed while neuronal white matter integrity was studied using diffusion tensor imaging (DTI). For MRS, ^1^H-single voxel spectra were acquired for pre-specified separate volumes of interest (2 × 2 × 2 cm) in the Right Posterior White Matter (RPWM), anterior cingulate cortex (ACC) and Posterior Gray Matter (PGM) using point-resolved spectroscopy with automated shimming and water suppression (TE/TR/NS/Volume = 35/1500/128/8 cm^3^). These specific voxels have been used in several prior studies and have a representation of largely white matter (RPWM), gray matter (PGM) and those with both (ACC)^33^,^34^. DTI was performed using a single shot, spin-echo echoplanar imaging sequence (FOV = 256 mm, slice thickness = 2.0 mm, 70 contiguous axial slices, 128 × 128 matrix, TR = 9000 ms, TE = 80 ms, b-value = 1000 s/mm^2^, #b0 images = 6, #Diffusion Directions = 64).

#### MRI analysis

##### MRS

The choline (Cho), creatine (Cr), myo-inositol (mI) and glutamate + glutamine (Glx) complex peak areas were computed using LCModel software and their creatine ratios were used as in prior studies^35^,^36^,^37^. A high Glx and low mI and Cho creatine ratios are associated with cirrhosis and hyperammonemia. DTI: Whole brain voxel-wise maps of Fractional Anisotropy (FA), mean diffusivity (MD) and spherical isotropy (CS) were constructed from pre-processed DTI images using tools in FSL 5.0.2 (FMRIB’s Software Library, www.fmrib.ox.ac.uk/fsl)^38^,^39^,^40^,^41^. These maps were then transformed to standard space using a combination of nonlinear and affine registration tools. The *a priori* ROIs for major white matter tracts (Corpus callosum, internal capsule, inferior and superior longitudinal fasciculi, frontal and posterior white matter, uncinate fasciculi, insula and corticospinal tracts) were created using the DTI-based probabilistic white matter atlases^42^,^43^ using a probability threshold of 40%. Mean FA, MD and CS values were extracted from individual maps. A low FA and high CS and MD indicate interstitial edema.

#### Correlation network analysis

We created correlation networks using tools in the Galaxy Portal at the Microbiome Analysis Center and only included nodes consisting of microbiota, MELD score, cognitive tests and creatine ratios of mI, Cho and Glx in all three regions of interest which had a p value <0.01 and correlation coefficient of >0.6 or <−0.6. These networks were created for controls and cirrhotic subjects and then also within cirrhotic subjects into those with and without prior HE^44^. We then analyzed correlation differences between cirrhotics with and without HE and these were then visualized in Cytoscape^45^.

## Additional Information

**How to cite this article**: Ahluwalia, V. *et al*. Impaired Gut-Liver-Brain Axis in Patients with Cirrhosis. *Sci. Rep.* **6**, 26800; doi: 10.1038/srep26800 (2016).

## Supplementary Material

Supplementary Information

## Figures and Tables

**Table 1:** Demographics and details of enrolled subjects with and without cirrhosis.

Mean ± SD unless stated otherwise	Healthy controls (n = 40)	Cirrhosis (n = 147)	
		Without Prior HE (n = 62)	Prior HE (n = 85)
Age	55.1 ± 13.4	54.2 ± 11.6	56.2 ± 13.5
Gender (men/women)	23/17	46/16	16/69
Serum sodium	142 ± 6.8	137.9 ± 4.2^†^	134.7 ± 5.9***
Venous Ammonia	24.7 ± 14.6	41.2 ± 18.6^††^	57.5 ± 29.9***
24 hour caloric intake	2340 ± 462	2365 ± 737	2235 ± 389
Cirrhosis details			
Etiology (HCV/Alcohol/HCV+ Alcohol/NASH/Other)	–	21/15/3/15/8	27/16/20/12/10
MELD score	–	11.0 ± 4.2	15.5 ± 5.2***
Lactulose use	–	–	85 (100%)
Rifaximin use	–	–	52 (62%)
Inflammatory markers			
Endotoxin (EU/ml)	0.1	1.2^††^	3.4***
IL-1b (pg/ml median)	0.0	0.0	0.7*
IL-6 (pg/ml median)	0.0	0.0	6.2***
TNF(pg/ml median)	3.5	5.7	8.2*
Cognitive tests			
Number connection-A (seconds)	24.6 ± 20.5	35.3 ± 11.8^†^	53.2 ± 34.8***
Number connection-B (seconds)	79.1 ± 46.1	89.4 ± 33.7	165 ± 121***
Digit symbol (raw score)	81.1 ± 14.2	57.3 ± 12.5^†^	43.0 ± 17.3***
Block design (raw score)	43.3 ± 16.1	28.7 ± 12.5^†^	20.8 ± 12.6***
Line tracing time (seconds)	101.2 ± 58.6	106.5 ± 46.5	137 ± 101*
Line tracing time (errors)	27.3 ± 25.2	31.2 ± 29.8	44.0 ± 35.7*
Serial dotting time (seconds)	46.3 ± 21.2	64.3 ± 24.7^†^	91.7 ± 24.7***
ICT lures (no. responded)	6.7 ± 4.3	10.5 ± 8.3	15.5 ± 9.1**
ICT targets (% correct)	97.8 ± 5.7	97.3 ± 3.7	88.1 ± 11.6***

*p < 0.05, ***p < 0.0001 in cirrhosis with HE compared to other groups, ^††^p < 0.01, ^†^p < 0.05 in cirrhosis without HE compared to controls; A high score on Digit symbol and Block design tests and low value on the other tests indicate good cognitive performance^†††^.

**Table 2:** Changes in Brain MRI values (only significant changes on DTI are shown).

MR spectroscopic values	Cirrhosis without prior HE (n = 62)	Cirrhosis with prior HE (n = 85)
Parietal White Choline	0.28 ± 0.07	0.25 ± 0.05*
Parietal White Glx	2.12 ± 0.64	2.79 ± 0.81***
Parietal White mI	0.61 ± 0.36	0.31 ± 0.25***
Posterior Gray Choline	0.19 ± 0.03	0.18 ± 0.05
Posterior Gray Glx	2.29 ± 0.64	2.80 ± 0.73***
Posterior Gray mI	0.62 ± 0.27	0.39 ± 0.20***
Anterior Cingulate Choline	0.26 ± 0.04	0.23 ± 0.04*
Anterior Cingulate Glx	2.57 ± 0.64	3.23 ± 0.88***
Anterior Cingulate mI	0.61 ± 0.27	0.37 ± 0.22***
**Diffusion tensor imaging (DTI)**	**Cirrhosis without prior HE (n = 31)**	**Cirrhosis with prior HE (n=42)**
Spherical Isotropy		
L Anterior Internal Capsule	0.48 ± 0.04	0.51 ± 0.02*
R Anterior Internal Capsule	0.47 ± 0.03	0.49 ± 0.03*
Corpus callosum genu	0.46 ± 0.05	0.48 ± 0.04*
Corpus callosum body	0.42 ± 0.05	0.45 ± 0.05*
Corpus callosum splenium	0.32 ± 0.04	0.34 ± 0.03*
R External Capsule	0.60 ± 0.03	0.62 ± 0.01*
L Frontal White	0.59 ± 0.03	0.61 ± 0.03*
R Posterior Internal Capsule	0.38 ± 0.2	0.40 ± 0.03*
R Superior Longitudinal Fasciculus	0.54 ± 0.03	0.58 ± 0.02*
Fractional Anisotropy		
L Anterior Internal Capsule	0.58 ± 0.03	0.56 ± 0.02*
R Anterior Internal Capsule	0.60 ± 0.03	0.58 ± 0.03*
Corpus callosum genu	0.66 ± 0.03	0.64 ± 0.04*
Corpus callosum body	0.69 ± 0.04	0.66 ± 0.04*
Corpus callosum splenium	0.76 ± 0.02	0.74 ± 0.05*
R Cingulum	0.53 ± 0.03	0.50 ± 0.03*
L External Capsule	0.43 ± 0.03	0.41 ± 0.02*
R External Capsule	0.42 ± 0.03	0.31 ± 0.02*
L Frontal White	0.47 ± 0.03	0.44 ± 0.03*
R Frontal White	0.48 ± 0.03	0.46 ± 0.03*
L Posterior Internal Capsule	0.68 ± 0.02	0.66 ± 0.03*
R Posterior Internal Capsule	0.68 ± 0.02	0.66 ± 0.02*

*p < 0.05, ***p < 0.0001, All spectroscopic values are ratios of creatine, Glx: glutamate + glutamine, mI: myoinositol, R: right, L: left, HE: hepatic encephalopathy. No changes on mean diffusivity were seen between groups.

**Table 3:** Differences in human stool microbiota composition.

% relative abundance	Controls (n = 40)	Cirrhosis without prior HE (n=62)	Cirrhosis with prior HE (n = 85)
Phylum (Mean ± SD)			
*Firmicutes*	50.5 ± 13.0	28.0 ± 19.0^†^	24.0 ± 20.0*
*Bacteroidetes*	33.0 ± 29.7	33.0 ± 30.0	24.0 ± 26.0*
*Proteobacteria*	1.4 ± 3.0	3.0 ± 6.0^†^	7.0 ± 15.0*
Phylum_Family (Mean ± SD)			
*Bacteroidetes_Bacteroidaceae*	21.0 ± 15.9	22.2 ± 24.0	18.2 ± 25.2*
*Bacteroidetes_Porphyromonadaceae*	5.0 ± 8.7	2.1 ± 5.2	3.9 ± 7.7*
*Bacteroidetes_Prevotellaceae*	4.7 ± 14.7	4.5 ± 13.5	2.9 ± 8.0*
*Bacteroidetes_Rikenelleaceae*	2.2 ± 2.6	2.0 ± 5.5	0.0 ± 2.4*
*Firmicutes_Lactobacillaceae*	0.0 ± 1.7	2.0 ± 8.5^†^	4.0 ± 9.5*
*Firmicutes_Enterococcaceae*	0.0 ± 0.0	1.1 ± 6.7^†^	3.6 ± 10.9*
*Firmicutes_Clostridiales XIV*	8.7 ± 5.9	3.2 ± 4.7^†^	1.4 ± 3.5*
*Firmicutes_Lachnospiraceae*	23.7 ± 7.9	10.5 ± 8.4^†^	6.3 ± 11.0*
*Firmicutes_Ruminococcaceae*	13.4 ± 6.9	5.5 ± 6.2	3.0 ± 5.4*
*Firmicutes_Veillonellaceae*	2.2 ± 3.2	1.7 ± 2.9	2.0 ± 4.8
*Proteobacteria_Enterobacteriaceae*	0.0 ± 0.0	2.4. ± 5.9^†^	5.5 ± 14.8*

*p < 0.05 in HE subjects compared to controls and those without HE, ^†^p < 0.05 in cirrhosis without HE compared to controls; HE: hepatic encephalopathy.
