# Supplementary material for: Impaired Gut-Liver-Brain Axis in Patients with Cirrhosis
Source: Sci Rep. 2016 May 26;6:26800. doi: 10.1038/srep26800 (PMC4880966; doi:10.1038/srep26800)
Supplement: Supplementary Information [file srep26800-s1.pdf]

Impaired Gut-Liver-Brain Axis in Patients with Cirrhosis

Vishwadeep Ahluwalia[1], Naga S Betrapally[2], Phillip B Hylemon[3], Melanie B White[1], Patrick M Gillevet[2], Ariel B Unser[1], Andrew Fagan[1], Kalyani Daita[1], Douglas M Heuman[1], Huiping Zhou[3], Masoumeh Sikaroodi[2], Jasmohan S Bajaj[1]

[1]Gastroenterology, Hepatology and Nutrition, [3]Microbiology and Immunology, Virginia Commonwealth University and McGuire VA Medical Center, Richmond, Virginia [2]Microbiome Analysis Center, George Mason University, Manassas, Virginia

Supplementary figure legends

Figure S1: PiCRUST analysis of predicted bacterial functions.

S1A:Human stool analysis of controls vs. cirrhotics. Red=cirrhosis, Green=controls. This shows a significantly increased predicted bacterial functions related to endotoxin and endotoxin protein synthesis as well as aromatic amino acid metabolism in cirrhotic stool.

S1b: Human stool analysis of cirrhotic patients. Red=hepatic encephalopathy, Green=no hepatic encephalopathy. There was a significantly higher aromatic amino acid metabolism-related predicted function in stool microbiota of patients with hepatic encephalopathy

S1A

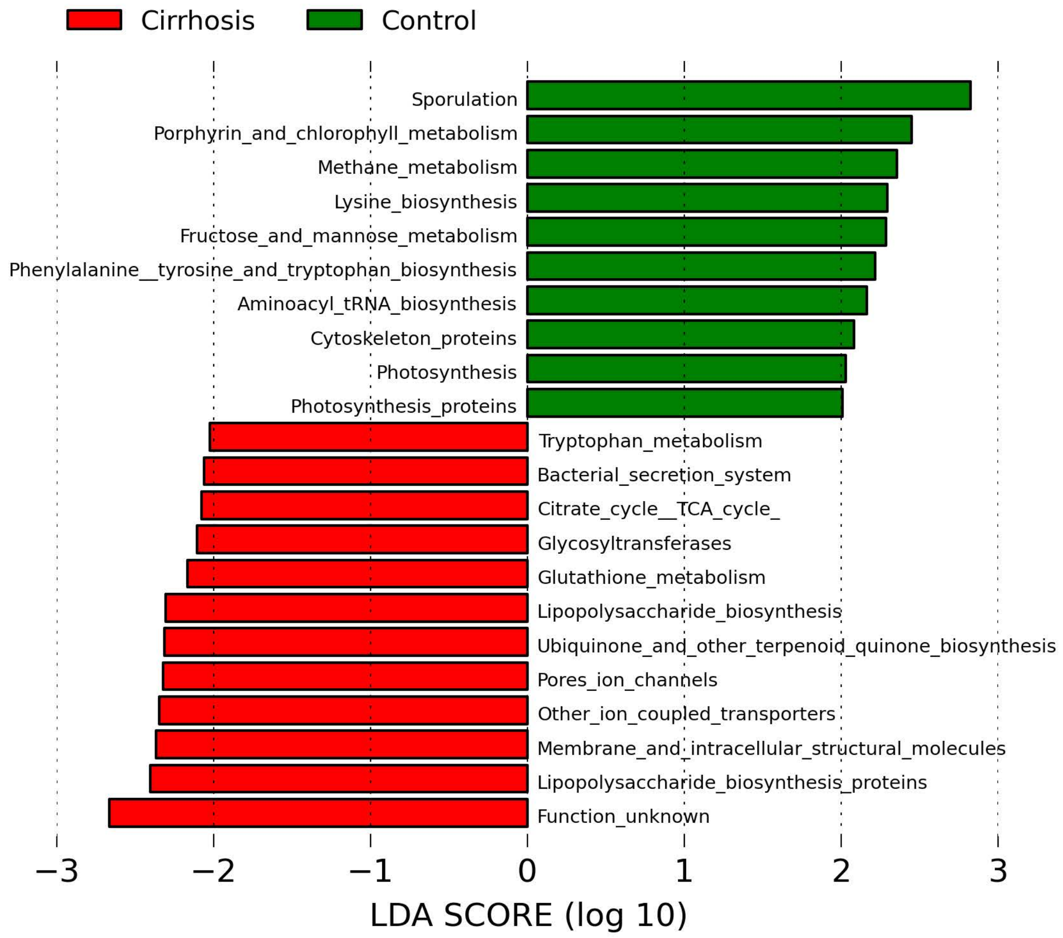
Cirrhosis
Control
Sporulation
Porphyrin_and_chlorophyll_metabolism
Methane_metabolism
Lysine_biosynthesis
Fructose_and_mannose_metabolism
Phenylalanine__tyrosine_and_tryptophan_biosynthesis
Aminoacyl_tRNA_biosynthesis
Cytoskeleton_proteins
Photosynthesis
Photosynthesis_proteins
Tryptophan_metabolism
Bacterial_secretion_system
Citrate_cycle__TCA_cycle_
Glycosyltransferases
Glutathione_metabolism
Lipopolysaccharide_biosynthesis
Ubiquinone_and_other_terpenoid_quinone_biosynthesis
Pores_ion_channels
Other_ion_coupled_transporters
Membrane_and_intracellular_structural_molecules
Lipopolysaccharide_biosynthesis_proteins
Function_unknown
−3
−2
−1
0
1
2
3
LDA SCORE (log 10)

S1B

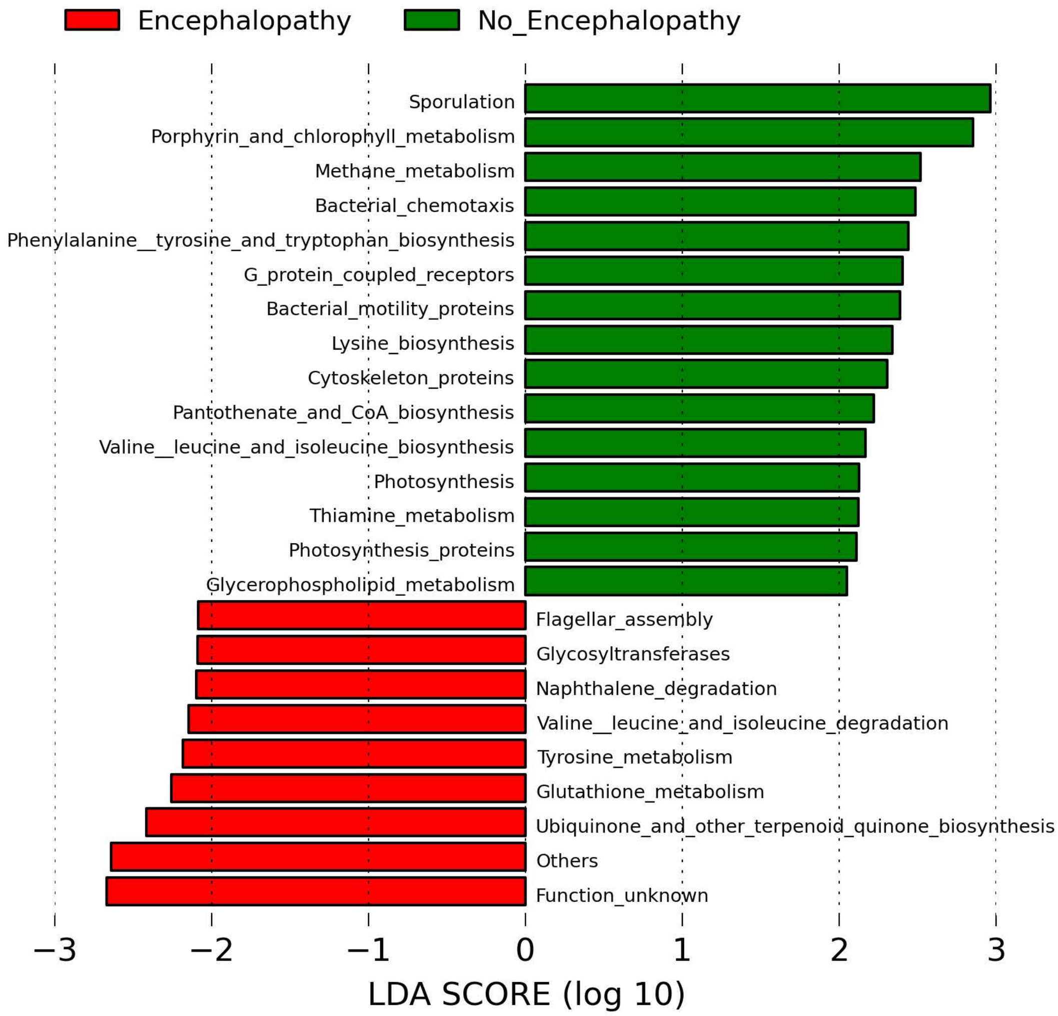

Encephalopathy
No_Encephalopathy
Sporulation
Porphyrin_and_chlorophyll_metabolism
Methane_metabolism
Bacterial_chemotaxis
Phenylalanine__tyrosine_and_tryptophan_biosynthesis
G_protein_coupled_receptors
Bacterial_motility_proteins
Lysine_biosynthesis
Cytoskeleton_proteins
Pantothenate_and_CoA_biosynthesis
Valine__leucine_and_isoleucine_biosynthesis
Photosynthesis
Thiamine_metabolism
Photosynthesis_proteins
Glycerophospholipid_metabolism
Flagellar_assembly
Glycosyltransferases
Naphthalene_degradation
Valine__leucine_and_isoleucine_degradation
Tyrosine_metabolism
Glutathione_metabolism
Ubiquinone_and_other_terpenoid_quinone_biosynthesis
Others
Function_unknown
−3
−2
−1
0
1
2
3
LDA SCORE (log 10)
